# Supplementary material for: Tuning site-specific dynamics to drive allosteric activation in a pneumococcal zinc uptake regulator
Source: eLife. 2018 Oct 17;7:e37268. doi: 10.7554/eLife.37268 (PMC6224198; doi:10.7554/eLife.37268)
Supplement: Supplementary file 1. [file elife-37268-supp1.docx]

**Supplementary Data**

**Tuning site-specific dynamics to drive allosteric activation in a pneumococcal zinc uptake regulator**

Daiana A. Capdevila,^†^ Fidel Huerta,^†,‡^ Katherine A. Edmonds,^†^ My T. Le,^†^ Hongwei Wu,^†^ and David P. Giedroc*^,†,§^


This file contains **Supplementary Tables S1-S2**

**Table S1.** Zinc binding affinities of wild-type AdcR and selected AdcR mutants characterized here.

|  | Zn^II^ binding to site 2 in the homodimer | |
| --- | --- | --- |
| AdcR | *K*_Zn, 3_  (x10^9^ M^-1^) | *K*_Zn, 4_  (x10^9^ M^-1^) |
| wild-type | ≥1 | 0.0205 ±0.0013 |
| V34A | 0.0022±0.0017 | (9.4±8.2) 10^-5^ |
| L81V | ≥1 | 0.025±0.0027 |
| L57M | ≥1 | 0.0169±0.001 |
| L57V | ≥1 | 0.119±0.018 |
| I16A | ≥1 | 0.00479±0.0005 |
| V142A | 0.00085±0.00018 | <10^-5^ |
| L17A | ≥1 | .00158±0031 |
| I27A | ≥1 | .00349±.005 |

^a^Conditions: 10 mM Hepes, pH 7.2, 0.4 M NaCl, 1 mM TCEP (chelexed), 15 μM Mf2, 25.0 ºC titrated with ZnCl_2_ solutions. Experiments were conducted 3 times for each AdcR variant. Errors of the binding constant parameters were estimated from global fits.

^b^*K*_Zn,1_ and *K*_Zn,2_ were fixed to a value of 1 x10^12^ M^-1^. ^c^ *K*_Zn,MF2_ = (4.9 ± 0.6) x10^6^ M^-1^ under these solution conditions.

**Table S2.** Differential Scanning Fluorimetry with SYPRO Orange

|  | | Apo | |  | | Zn^II^ |
| --- | --- | --- | --- | --- | --- | --- |
| AdcR | pH 7.0^a^  T_m_ (^o^C) | | pH 5.5^b^  T_m_ (^o^C) | | pH 7.0  T_m_ (^o^C) | |
| wild-type | 47±1 | | 45±1 | | 69±1 | |
| I16A | 45±1 | | _ | | _ | |
| L17A | 52±1 | | _ | | 71±1 | |
| I27A | 49±1 | | _ | | 66±1 | |
| V34A | 46±1 | | 36±1 | | 70±1 | |
| L57V | 46±1 | | _ | | 66±1 | |
| L57M | 46±1 | | 45±1 | | 67±1 | |
| L61V | 37±1 | | _ | | _ | |
| V63A | 41±1 | | _ | | 64±1 | |
| L81V | 51±1 | | _ | | _ | |
| V142A | 49±1 | | _ | | 67±1 | |

^a^Conditions: 10 mM Hepes, pH 7.0, 0.23 M NaCl, 1 mM TCEP (chelexed), with 4 μM protein, 5x SYPRO orange, 10 µM EDTA (For apo AdcRs) or 2 protomer mol•equivalents of ZnCl_2_ (for Zn^II^_2_ AdcR) added to these reactions. ^b^25mM MES, pH 5.5, 50 mM NaCl, 1 mM TCEP (chelexed); all other conditions the same.
